# Prevention of retinal light damage by zinc oxide combined with rosemary extract

**Published:** 2013-06-27

**Authors:** Daniel T. Organisciak, R. M. Darrow, C. M. Rapp, J.P. Smuts, D.W. Armstrong, J. C. Lang

**Affiliations:** 1 Petticrew Research Laboratory, Department of Biochemistry and Molecular Biology, Wright State University Boonshoft School of Medicine, Dayton, OH; 2 Department of Chemistry and Biochemistry, University of Texas at Arlington, Arlington, TX

## Abstract

**Purpose:**

Zinc oxide effectively reduces visual cell loss in rats exposed to intense visible light and is known to slow the rate of disease progression in advanced stages of age-related macular degeneration. Our goal was to determine the efficacy of zinc oxide in combination with novel and well-established antioxidants in an animal model of light-induced oxidative retinal damage.

**Methods:**

One group of male Sprague-Dawley rats was pretreated with zinc oxide with or without a detergent extract of rosemary powder and then exposed to intense visible light for 4–24 h. Another group of animals received zinc oxide combined with rosemary oil diluted with a mixture of polyunsaturated fatty acids (ROPUFA) and a third group was given an antioxidant mineral mix containing zinc oxide, as recommended by the Age Related Eye Disease Study group's first clinical trial (AREDS1). Visual cell survival was determined 2 weeks after intense light treatment by measuring rhodopsin and photoreceptor cell DNA levels and confirmed by retinal histology and agarose gel electrophoresis of DNA. Western analysis was used to determine the effects of zinc and antioxidants on the oxidative stress markers, glial fibrillary acidic protein (GFAP), heme-oxygenase-1 (HO-1), and carboxyethylpyrrole (CEP). Rod and cone opsin and arrestin levels were used as markers of photoreceptor cell function.

**Results:**

Dark-reared rats treated with 1.3 mg/kg zinc oxide and 17 mg/kg rosemary extract, or with one-half those doses, and exposed to moderate intensity green light retained 75%–85% of the rhodopsin and retinal DNA measured in unexposed rats. These levels were significantly higher than found for zinc oxide or rosemary treatment alone. Rosemary oil was also effective when combined with zinc oxide, but ROPUFA alone was no more effective than the detergent vehicle. Prolonged intense green light led to increases in retinal GFAP and HO-1 levels and to decreases in cone cell opsin and rod and cone arrestins. Rosemary plus zinc treatment reduced the expression of oxidative stress protein markers and enhanced visual cell survival, as shown by improved photoreceptor cell morphology and by decreased retinal DNA degradation. Using higher intensity white light for exposures in cyclic light-reared rats, treatment with an AREDS antioxidant/mineral mixture was found to be ineffective, whereas rosemary extract plus an equivalent dose of zinc oxide was significantly more effective in preserving visual cells. CEP protein adduct formation was reduced by all antioxidant treatments, but rosemary plus zinc oxide also prevented the loss of cone cell opsin and arrestin more effectively than AREDS.

**Conclusions:**

In the rat model of acute retinal light damage, zinc oxide combined with a detergent extract of rosemary powder or rosemary oil is more effective than treatment with either component alone and significantly more effective than an AREDS mixture containing a comparable dose of zinc oxide. Light-induced oxidative stress in animal models of retinal degeneration can be a useful preclinical paradigm for screening novel antioxidants and for testing potential therapeutics designed to slow the progression of age-related ocular disease.

## Introduction

Age-related macular degeneration (AMD) is a multifactorial disease with a strong genetic component involving the complement system [1-4] and an increased risk of vision loss associated with oxidation and inflammation [4,5]. Epidemiology indicates that while age is the primary risk factor for vision loss, smoking-induced oxidative stress increases that risk at least twofold [5-9]. Proteomic evidence for oxidative protein damage has been found in drusen-isolated postmortem from the Bruch’s membrane-choroid retinal pigment epithelium (RPE) complex [10]. Drusen from patients with AMD were found to contain higher levels of oxidized lipid-protein adducts and α-crystallins than found in normal age-matched controls [10,11]. A related risk factor is a low serum antioxidant index [5,12]. In the Age Related Eye Disease Study (AREDS), the rate of disease progression in intermediate to advanced AMD was reduced for individuals taking an antioxidant/mineral mix containing vitamins A (as β-carotene), C, and E along with zinc- and cupric-oxide [12]. Treatment with the AREDS vitamin/mineral mix led to a decrease in the rate of neovascularization in approximately one-quarter of advanced cases, but zinc appears to have accounted for most of the benefit [12]. Zinc is known to enhance binding of complement factor H (CFH) to factor C3B, leading to suppression of complement-mediated cell lysis [8,13]. CFH also binds epitopes on proteins modified by malondialdehyde, a byproduct of fatty acid oxidation, leading to an inhibition of further oxidative damage and to a decrease in pro-inflammatory responses [14].

Antioxidants and the trace element zinc also are known to decrease oxidative stress and to prolong photoreceptor cell life in animal models of retinal degeneration. Numerous antioxidants are capable of reducing both the extent of tissue damage and visual cell loss in the light-induced retinal degeneration model, providing compelling evidence that implicates oxidative damage [15]. Using dose –response curves to determine relative antioxidant efficacy, detergent extracts of the herb rosemary (*Rosmarinus officinalis*) were found to be approximately ten times more effective in preventing retinal light damage than vitamin C [16]. Rosemary is known to contain a complex mixture of phenolic antioxidants, including carnosol, carnosic acid, rosmanol, rosmarinic acid, and ursolic acid [17-20]. Recently, pretreatment of rats [21] with zinc oxide at or above AREDS recommended levels was found to decrease retinal light damage and to increase visual cell survival [22]. Similarly, in an animal model of diabetic retinopathy, daily administration of an AREDS-based micronutrient mix was found to decrease oxidation and retinal pathology [23,24].

The mosaic arrangement of rod and cone photoreceptors in rodent retina differs from that in primates, limiting our ability to extrapolate between species. However, some aspects of AMD etiology can be replicated in rodent animal models [25]. For example, Hollyfield and associates [26] immunized mice with carboxyethylpyrrole (CEP) adducted to mouse serum albumin and found inflammatory changes in Bruch’s membrane along with the accumulation of drusen. Importantly, CEP is formed from oxidation of the 22:6 n-3 fatty acid docosahexaenoic acid (DHA), found in abundance in photoreceptor cells and as oxidized protein adducts in drusen [10]. In a related study, a mouse model with a superoxide dismutase (SOD) gene deletion was found to exhibit age-dependent pathological changes in Bruch’s membrane and RPE, changes exacerbated by excessive light [27]. Using a rat model of acute light-induced phototoxicity, retinal microglia activation and migration [28] along with macrophage invasion [29] were found to stimulate the alternative complement pathway and to mediate deposition of factor C3, factor B, and CFH in the area of photic lesions [28,29]. Finally, Marc et al. [30] compared end stage morphology in light-damaged rat retina with changes in late-stage atrophic AMD and found remarkable anatomic similarities in postdamage retinal remodeling.

As a model of oxidative stress and to test the protective efficacy of various antioxidant and zinc combinations, we used the rat model of light-induced retinal degeneration. We found that rosemary extract emulsified with detergent or with a mixture of polyunsaturated fatty acids and detergent, and aqueous zinc oxide were each effective in reducing retinal damage and in increasing visual cell survival. The protective effect for rosemary combined with zinc was greater than for either compound alone and greater than for the combined ingredients in a typical AREDS vitamin/mineral mixture [12]. The use of zinc oxide combined with phenolic antioxidants may provide new insights into the mechanism(s) of oxidative tissue damage and suggests new possibilities for therapies designed to slow or prevent the progression of AMD.

## Methods

Animals, rearing conditions, intense light treatment: Male Sprague-Dawley rats were obtained from Harlan Inc. (Indianapolis, IN) as weanlings and allowed to acclimate to darkness or to dim cyclic light for a period of 40 days. The dark environment was interrupted for less than 30 min each day for routine animal maintenance. During these periods dim red light (>600 nm) was used to illuminate the room. The cyclic light environment consisted of 12 h white light–12 h dark per day, with light provided by 7 W “night lights” affixed to the cage racks in multiple outlet strips above the polycarbonate animal cages. Light intensity was regulated by adjusting line voltage to provide a dim light environment of 10–20 lux, and lights were on each day at 8 AM. All animals were fed Rat Chow (Teklad, Madison, WI) and given water ad libitum.

At P60-P65, rats were exposed to intense visible light using one of two testing paradigms. Dark-reared rats were treated for 4 h in circular green Plexiglas #2092 (GE Polymer Shapes, Dayton, OH) chambers with 490–580 nm light [31] at an intensity of 1,200 lux. Some dark-reared animals were exposed to light for as long as 24 h. Cyclic light-reared rats were exposed to full spectrum white light (400–700 nm) in clear Plexiglas chambers for 6 h at an intensity of 9,000 lux. For either light exposure paradigm, two rats were treated in each chamber and all exposures started at 9 AM. For antioxidant treatment, rats were dark adapted overnight and then given an intraperitoneal (IP) injection 1 h before the start of light exposure. Rats treated with vehicle were also dark adapted and then exposed to intense visible light, normally in the same chambers as antioxidant-treated animals. Following light, all rats were moved to darkness for 1–2 days for protein- and DNA- gel electrophoresis or for 14 days to assess visual cell survival. Rats were killed in a CO_2_-saturated atmosphere under dim red illumination. The use of animals in this investigation conformed to the Association for Research in Vision and Ophthalmology statement for the Use of Animals in Ophthalmic and Vision Research, and the Laboratory Animal Care and Use Committee guidelines at Wright State University.

### Antioxidants

Rosemary powder was provided by LycoRed Ltd. (Beer Sheva, Israel) and obtained from Alcon Ltd. (Fort Worth, TX). The powder was used at a concentration of 25 mg/ml. It was first wetted with 100% ethanol (0.1 ml/25 mg), flushed with argon, capped, and kept in darkness for 2 h at room temperature. The slurry was then brought to volume with 1% aqueous Tween-80 (Sigma, St. Louis, MO), sonicated (2×10 s bursts) at 80% setting (Biosonic; Bronwell Scientific, Rochester, NY), argon flushed, capped, vortexed, and kept at 4 °C overnight. The following morning the mixture was again vortexed, and an appropriate volume of the uniformly emulsified suspension was injected IP into experimental animals.

Rosemary oil extract solubilized in a mixture of omega-3 polyunsaturated fatty acid ethyl esters (ROPUFA; 75 N-3 EE) from DSM (Kaiseraugst, Switzerland) was also provided by LycoRed Ltd. and obtained from Alcon. The solubilized mixture (RSEE1030061) was diluted with 1% aqueous Tween-80/10% ethanol (vehicle), emulsified by vortexing, and used immediately after preparation. A dose–response was determined by giving increasing amounts of the rosemary oil extract to rats by IP injection. Storage of both rosemary oil and ROPUFA was at 4 °C under argon. The levels of rosemary active in the powder and oil preparations are reported in the Supplemental Information (Appendix 1) accompanying this paper. Zinc oxide (ZnO) was purchased from Alfa Aesar (Ward Hill, MA) and dissolved in acidified water (pH 2) for use at a concentration of 2 mg/ml.

A commercial AREDS1 antioxidant/mineral formulation (ICAPS, Alcon Ltd.) was obtained locally. The contents of 1 softgel capsule were brought to 25 ml with 1% Tween-80 containing 16% ethanol and 20% ROPUFA. The mixture was then vortexed and the emulsion used immediately. AREDS-recommended daily doses [12] for a 60-kg individual for vitamins C and E, β-carotene, zinc oxide, and copper are: 7.53; 6.67; 0.29; 1.44, and 0.027 mg/kg, respectively (Appendix 1). The AREDS detergent/ROPUFA extract was administered by IP injection and given at a level that approximated the daily recommended dose of zinc.

Photoreceptor cell survival and antioxidant efficacy: To assess the capacity of antioxidant mixtures to prevent light-induced photoreceptor cell loss, we routinely measured rhodopsin and retinal DNA levels 2 weeks after intense light treatment. For these determinations, rhodopsin was measured in one eye of each rat and retinal DNA in the other. Rhodopsin was determined in Emulphogene BC-720 (Sigma) detergent extracts as described [21,22]. The decrease in 500 nm absorbance measured first in darkness and then after bleaching experimental sample extracts was compared with that from unexposed control rat eyes. From these measurements photoreceptor cell survival was calculated as n mol rhodopsin per eye. Similarly, retinal DNA, measured with the Hoechst (Calbiochem-Behring, La Jolla, CA) dye-binding assay [21,22], was compared in experimental and control rat retinas. Photoreceptor cell DNA was then determined by subtracting the DNA content of the inner retinal layers and is presented as μg DNA per retina. Protective efficacy for the various antioxidants was determined from the averages for visual cell survival following light damage. It is based on the respective retention of rhodopsin and photoreceptor cell DNA in experimental animals and in those treated with vehicle and exposed to intense visible light [22].

### Histology

Rat eyes were enucleated 2 weeks after light treatment and oriented so the superior hemisphere could be marked to insure identification during tissue processing. The lens was excised and the eyecups fixed in 50% Karnovsky’s solution for 24 h. Tissues were then transferred to 0.1 M sodium cacodylate buffer, pH 7.4, and kept at 4 °C. Eyecups were paraffin embedded, using a Tissue-Tek Vacuum Infiltration Processor (Sakura Finetek, Torrance, CA), and sectioned vertically at 4 µm, using a Shandon Finesse Microtome (Thermo Fisher, Rockford, IL). All sections were stained with hematoxylin and eosin, and the mid-superior region of the retina was examined.

### Gel electrophoresis of retinal proteins and DNA

For western blot analysis of proteins, dark-reared rats (n=3) were exposed to green light for either 4 or 24 h. Cyclic light-reared rats (n=4) were exposed to white light for 6 h. All animals were sacrificed 48 h after the start of light treatment and retinas excised using the basic technique of Winkler [32]. Briefly, eyes were proptosed using a full curved forceps, the cornea cut and lens and vitreous removed, after which the retina was extruded from the eye cup by gently raising the forceps. Protein concentrations were determined by the Bradford method. Tissue homogenates were mixed with sample buffer and kept at 4 °C for 30 min. Retinal proteins (20 μg/lane) were then electrophoresed through a 4% stacking–12.5% running gel, using a Mini Protean 3 apparatus (Bio-Rad Laboratories, Hercules, CA). Electrophoresis was conducted for 30 min at 35 V then 80 V for 1.5 h. Following electrophoresis, proteins were transferred overnight onto polyvinylidene fluoride (PVDF) membranes at 200 mA in 10 mM 3-(cyclohexylamino)-1-propane sulfonic acid (CAPS; pH 11) containing 20% methanol. Western blots were blocked in phosphate buffered saline (PBS; Sigma-Aldrich, St. Louis, MO) containing 2% nonfat dry milk and 0.2% Tween-20 for 1 h at 37 °C. Blots were then probed with primary antibody for 1 h at 37 °C, washed three times for 5 min in 0.1× blocking buffer, incubated with horseradish peroxidase (HRP) secondary antibody (Bio-Rad; 1:3,000) for 1 h at 37 °C, and rewashed three times for 5 min in 0.1× blocking buffer and then once for 5 min in PBS. Western blots were visualized by chemiluminescence and exposure to autoradiographic film. A summary of the primary antibodies used in this work and their dilutions is contained in Table 1.

**Table 1:** Primary antibodies used for western analysis of retinal proteins

Protein	Dilution	Source
Glyceraldehyde-P-dehydrogenase(GAPDH)	1:2000	Enzo Life Sciences, Plymouth Meeting, PA
Heme-oxygenase (HO-1)	1:1000	Enzo Life Sciences, Plymouth Meeting, PA
Glial fibrillary acidic protein (GFAP)	1:1000	Invitrogen, Carlsbad, CA
Carboxyethylpyrrole (CEP)	1:500	J. Crabb Cole Eye Inst. Cleveland, OH
Cone opsin, red/green	1:1000	Millipore, Temecula, CA
Cone arrestin (mCAR)	1:2000	C. Craft, USC, Los Angeles, CA
Rod opsin (rhodopsin)	1:5000	K.Palczewski, Case West Res. Cleveland, OH
S-antigen (S-ag) arrestin	1:2000	L. Donoso, Wills Eye Hosp. Philadelphia, PA

To determine the effects of antioxidants and zinc on DNA fragmentation, we used neutral pH agarose gel electrophoresis. DNA was isolated from individual rat retinas, using a Sigma Gene Elute kit. The DNA from three retinas from three different rats per test group was combined and samples (1 µg/lane) electrophoresed through 1.5% agarose (pH 7) at 50 V for 30 min (Easy-Cast B3 gel apparatus; Owl Scientific, Woburn, MA). Apoptotic DNA ladders and higher molecular weight fragments were visualized with ethidium bromide and photographed under ultraviolet light.

### Statistical methods

A two-tailed *t* test for paired sets of data was used to evaluate differences in rhodopsin and DNA values between experimental and control rat retinas. p<0.05 was considered to be a significant difference.

## Results

### Rosemary and zinc combinations

**Figure 1:**
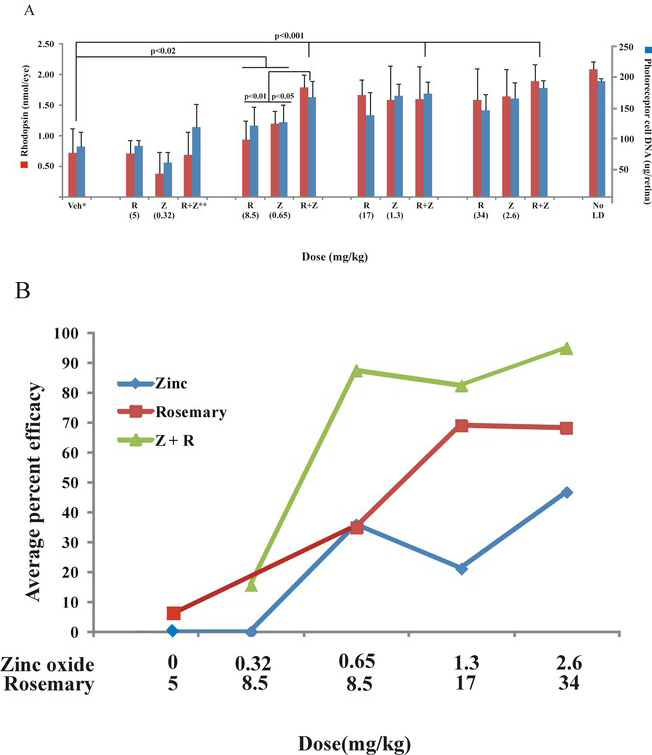
The protective effects of rosemary and zinc oxide on retinal light damage in dark-reared rats exposed to intense visible light. **A**: Rats were treated with various doses of rosemary powder extract (R) and or aqueous zinc oxide (Z) given 1 h before light exposure of 4 h duration and then dark maintained for 2 weeks before rhodopsin and photoreceptor cell DNA analysis. At doses of rosemary greater than 17 mg/kg and zinc oxide above 1.3 mg/kg, visual cell survival was significantly higher than for vehicle treatment, with no significant difference between the individual components and combined treatment (R+Z). At 8.5 mg/kg rosemary and 0.65 mg/kg zinc oxide, intermediate levels of visual cell rhodopsin and DNA were found for rosemary and zinc alone. These values were significantly higher than for vehicle but significantly lower than for combined rosemary plus zinc. At lower doses, neither rosemary nor zinc was effective. (*) Average rhodopsin and DNA values were combined for the 1% Tween-80/10% ethanol and acidified water vehicles. (**) Average values for rhodopsin and DNA measurements following treatment of rats with a combination of 0.32 mg/kg zinc oxide and 8.5 mg/kg rosemary powder extract. No LD represents data for rats unexposed to intense light. Results correspond to n=4-8 rats per treatment, ±standard deviation. **B**: The protective efficacy for pretreatment of rats with rosemary and zinc following exposure to intense visible light. Efficacy was calculated from the average values for rhodopsin and DNA in panel **A** (n=4-8) and plotted as average % for various doses of zinc oxide (blue diamond), rosemary extract (red square), and zinc plus rosemary (Z+R; green triangle). Efficacy was greater for the combined treatments than for either treatment alone over the entire dosing range. In this figure, and as described [21], percent efficacy was calculated using the following formula: 100x (nutrient treated - vehicle treated and light exposed) / (unexposed control - vehicle treated and light exposed).

Various concentrations of rosemary powder and zinc oxide were used to determine their protective effects on photoreceptor cell damage by light. Dark-reared rats (n=4–8/ treatment) were given a rosemary detergent extract as an emulsion, an aqueous acidic solution of zinc, oxide, or both (IP) and then treated with moderate intensity green light for 4 h. Following a 2-week dark recovery period, end-point analysis of visual cell survival was conducted by measuring rhodopsin and retinal DNA levels. Figure 1A shows that high levels of either rosemary or zinc were effective in preserving retinal photoreceptors. Using a detergent extract of rosemary powder at doses of 17 or 34 mg/kg, we found DNA and rhodopsin levels that were approximately 75% of those in unexposed dark-maintained controls. Zinc oxide at 1.3 or 2.6 mg/kg resulted in rhodopsin and DNA levels in the range of 80%–85% of control. At these higher doses, treatment of rats with combined rosemary plus zinc was nearly as effective as for the individual compounds. Decreasing the doses of rosemary and zinc oxide to 8.5 and 0.65 mg/kg, respectively, led to intermediate levels of visual cell survival. However, the average rhodopsin or retinal DNA levels in rosemary- or zinc-treated rats were still significantly higher (p<0.02) than for rats treated with vehicle. For combined treatment with rosemary and zinc, we found approximately 80% visual cell survival. Using this combined dose, rhodopsin and retinal DNA values were significantly higher than for rosemary or zinc alone (p<0.01; <0.05, respectively) or for the average of the Tween-80 detergent and acidified water vehicles (p<0.001). Further decreasing rosemary and zinc to 5.0 mg/kg and 0.32 mg/kg, respectively, resulted in their being largely ineffective. Zinc oxide combined with a slightly higher dose of rosemary (0.32 and 8.5 mg/kg) provided little additional protection.

Protective efficacies for rosemary powder and zinc oxide alone and in combination were calculated from the average rhodopsin and photoreceptor cell DNA values in experimental animals minus those in vehicle-treated rats [22]; results are plotted in Figure 1B. These results more accurately assess the effectiveness of drug treatment on visual cell survival when compared with the same markers in retinas from animals unexposed to intense light. As expected, the lowest dose of zinc or rosemary was ineffective, and rosemary combined with zinc resulted in only about 15% efficacy. However, approximately 35% protective efficacy was found when the doses of zinc oxide and rosemary were increased to 0.65 mg/kg and 8.5 mg/kg, respectively. With those combined doses, visual cell protection was nearly 85%. Zinc oxide plus rosemary at higher doses was equally effective (80%–90%), approximating the sum of the protective efficacies for each compound.

### Histology

**Figure 2:**
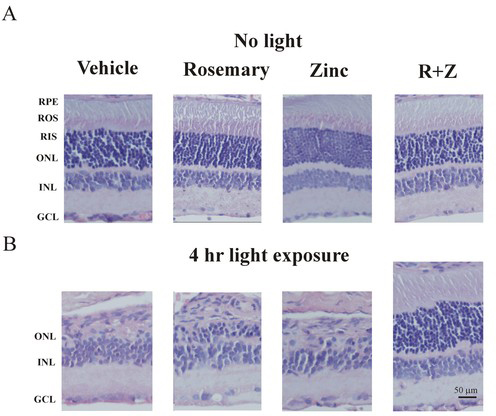
Retinal morphology in rats treated with rosemary extract and zinc oxide. Dark-reared animals were given intraperitoneal (IP) injections of rosemary (8.5 mg/kg), zinc oxide (0.65 mg/kg), or their combination (R+Z) and then exposed to green light for 4 h. Eyes were enucleated 2 weeks later and processed for histology. **A**: Vehicle, rosemary, and or zinc treatment had no effect on retinal morphology in dark-maintained control animals. The photoreceptor cell ONL contained eight to nine rows of nuclei, and ROS and RIS appeared normal. The INL and RPE were intact and also appeared to have normal morphology. **B**: Intense light exposure resulted in extensive ONL thinning in rats treated with vehicle. The ONL, containing only one to two rows of nuclei, appears opposed to the RPE, which is also damaged or missing. The INL is present in the space normally occupied by the ONL and an occasional misplaced nucleus appears in this section. Rosemary treatment resulted in the retention of three to four rows of photoreceptor cell nuclei in the ONL, whereas zinc treatment was largely ineffective. Rosemary plus zinc led to a much better outcome. The ONL contained eight to nine rows of nuclei, the INL and RPE were intact and their morphology appeared normal. The scale bar is equal to 50 µm.

To confirm the effectiveness of rosemary and zinc in preventing retinal damage, we compared their effects on photoreceptor cell morphology following light treatment. Rats were injected with rosemary alone (8.5 mg/kg), zinc oxide (0.65 mg/kg), or both and then treated with intense light or kept in darkness. Retinal histology was done 14 days later. As shown in Figure 2A, rosemary and/or zinc had no effect on retinal morphology in eyes from rats unexposed to light. The photoreceptor cell outer nuclear layer (ONL) contained eight to nine rows of nuclei. The rod inner segment (RIS) and outer segments (ROS), as well as the inner nuclear layer (INL) all appeared normal in both vehicle- and drug-treated animals. Four hours of intense green light, however, resulted in extensive photoreceptor cell loss in rats previously treated with vehicle (Figure 2B). The ONL consisted of only one to two rows of visual cell nuclei. Photoreceptor cell ROS and RIS were absent, and the RPE cell layer was damaged or missing. The INL was now present in the space normally occupied by the ONL, and some nuclei, apparently from the INL, appeared to be misplaced. Rosemary treatment resulted in better retinal morphology; there were three to four rows of nuclei in the ONL, and the INL appeared to be largely intact. Zinc treatment was less effective. The ONL was largely absent, and overall morphology resembled that seen in vehicle-treated animals. However, zinc combined with rosemary was very effective in preserving retinal morphology. The photoreceptor cell layer was intact, the RIS and ROS appeared normal, and the ONL contained eight to nine rows of nuclei. The RPE and INL were also largely intact and their morphology appeared normal.

### Electrophoretic analysis of DNA

**Figure 3:**
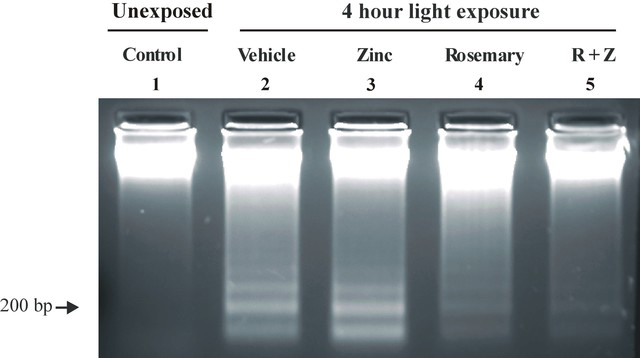
DNA electrophoresis following 4 h of moderate intensity green light. Retinal DNA was extracted from dark-reared rats (n=3) 48 h after the onset of light to assess the early response to toxic light exposure. DNA from three separate retinas was combined and then electrophoresed on a neutral pH agarose gel (1 μg/lane). High molecular weight DNA fragments and a 200-bp apoptotic ladder are clearly present in the vehicle- and zinc oxide (0.65 mg/kg) -treated rat retinas (lanes 2 and 3). Rosemary pretreatment (8.5 mg/kg) significantly reduced the appearance of a 200 bp apoptotic DNA ladder (lane 4). Zinc combined with rosemary (R+Z) was even more effective (lane 5). Retinal DNA appeared as a high molecular weight band near the top of the gel, resembling the pattern found for rats unexposed to intense light (lane 1).

In the rat, 71% of retinal DNA is contained in the ONL (20), implicating photoreceptor cell DNA damage and fragmentation in light-induced retinal degeneration. In light-exposed vehicle-treated rats, the electrophoretic pattern of retinal DNA exhibited a wide range of fragments of various molecular weights and a prominent 200 bp ladder indicative of apoptotic cell death (Figure 3, lane 2). Zinc oxide alone (0.65 mg/kg) had little beneficial effect as the pattern of DNA fragmentation resembled that found for vehicle-treated rats (lane 3). Rosemary treatment (8.5 mg/kg) was more effective. The staining intensity for low molecular weight DNA fragments was markedly reduced from that seen for vehicle or zinc treatment (lane 4 versus lanes 2, 3). Rosemary plus zinc was even more protective. The bulk of retinal DNA appeared in a high molecular weight band at the top of the gel (lane 5), resembling that seen for rats unexposed to intense light (lane 1), and a low molecular weight DNA ladder was barely detectable.

### Electrophoresis of retinal proteins

**Figure 4:**
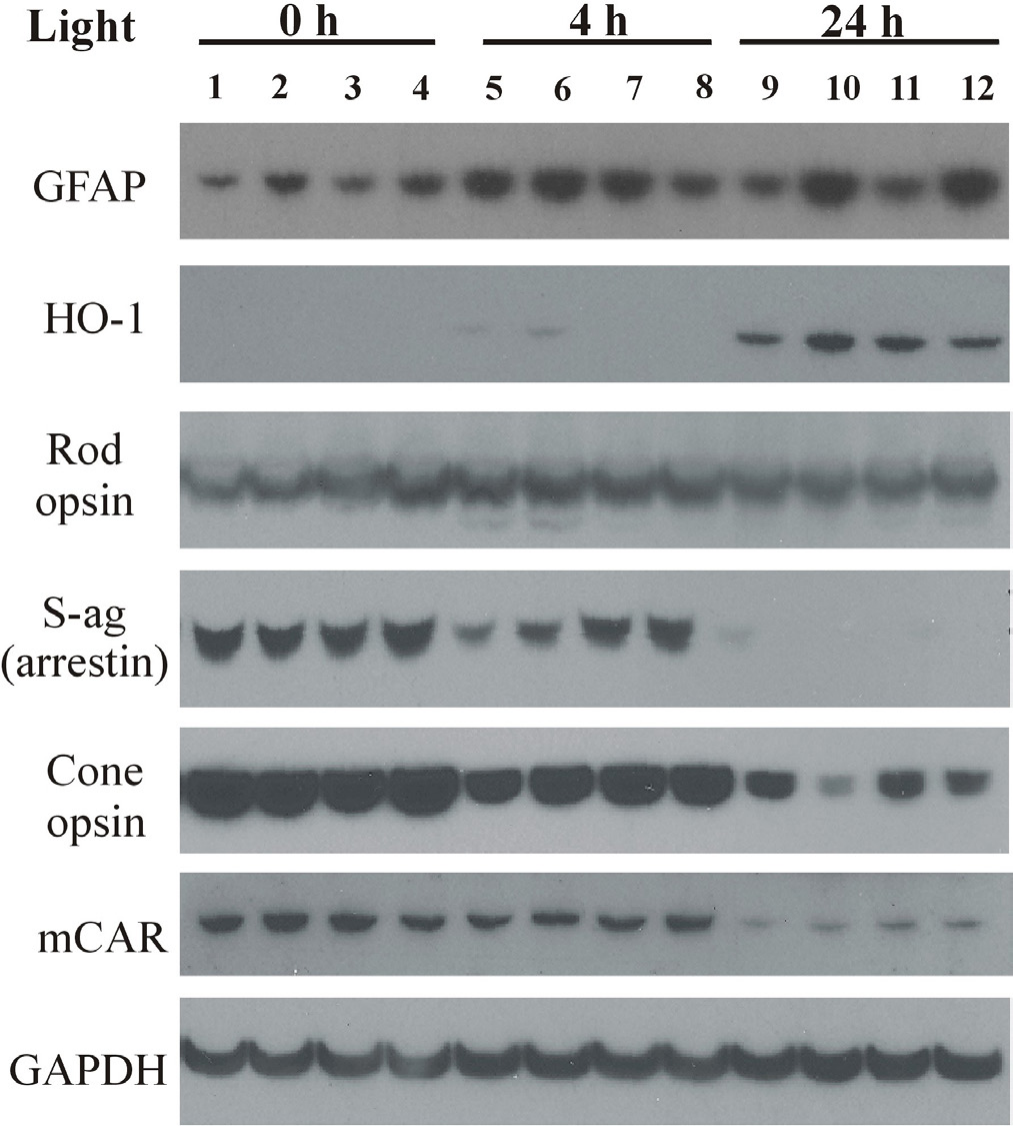
Western analysis following 0, 4, or 24 h of moderate intensity green light. Retinas were excised from dark-reared rats (n=3) unexposed to light (lanes 1–4), after 4 h of light exposure (lanes 5–8), or from those treated with 24 h of intense light (lanes 9–12). Retinal proteins from vehicle-treated animals are shown in lanes 1, 5, 9; zinc oxide (0.65 mg/kg)- treated in lanes 2, 6, 10; rosemary (8.5 mg/kg)- treated in lanes 3, 7, 11 and rosemary plus zinc treatment is shown in lanes 4, 8, 12. Proteins were extracted for 30 min at 4 °C and then electrophoresed (20 μg/lane) on a 12.5% polyacrylamide gel containing 1% sodium dodecyl sulfate (SDS) followed by transfer to polyvinylidene fluoride (PVDF) membranes and probing with primary antibodies (see Table 1). Staining for retinal glial fibrillary acidic protein (GFAP), a marker of Muller cell stress, was increased by intense light exposure of 4 h duration (lanes 5–8) and by 24 h intense light (lanes 9–12). Zinc treatment also resulted in GFAP induction (lanes 2, 6, 10), while rosemary appeared to reduce GFAP expression (lanes 3,7,11) compared to vehicle (lanes 1,5,9). Heme-oxygenase-1 (HO-1) levels were elevated by 24 h light exposure (lanes 9–12), while S-ag (rod arrestin), cone arrestin (mCAR), and cone opsin levels were all lower following 4 or 24 h of light. GAPDH staining indicates that protein loading per lane was relatively uniform.

Western analysis was used to assess the effects of light duration and antioxidant treatment on protein markers of oxidative stress and photoreceptor cell function. Figure 4 shows that the Muller cell protein glial fibrillary acidic protein (GFAP) was induced by intense green light. Although low GFAP staining was present in retinal extracts from rats unexposed to light (lanes 1–4), those from rats exposed to light for 4 h (lanes 5–8) or 24 h (lanes 9–12) had much higher levels. Zinc oxide (lanes 2, 6, 10) also appeared to induce GFAP expression compared with vehicle-treated animals (lanes 1, 5, 9), whereas immunoreactivity was approximately the same as vehicle for rats treated with rosemary alone (lanes 3, 7, 11). Increased retinal GFAP staining was present in extracts from two groups of rats given zinc plus rosemary (lanes 4, 12), while those treated with 4 h of light (lane 8) had reduced staining.

Heme-oxygenase-1 (HO-1) is a 32-kDa enzyme that is induced in retina by oxidative stress associated with intense visible light [33]. As expected, HO-1 immunoreactivity was absent in retinal extracts from rats unexposed to light (lanes 1–4); it was barely detectable in those treated with 4 h of light (lanes 5–8). However, 24 h of intense light resulted in HO-1 being present in all retinal extracts (lanes 9–12). HO-1 staining was slightly increased in retinas from zinc-treated animals (lane 10 versus 9) and approximately the same as vehicle for rats given rosemary alone or in combination with zinc (lanes 11, 12). The appearance of retinal HO-1 after 24 h of intense light (lanes 9–12) corresponded with only a modest decrease in rod cell opsin staining but with a dramatic loss of S-antigen (S-ag, rod arrestin). S-ag immunoreactivity was greater in extracts from rats treated with zinc and/or rosemary and 4 h of light (lanes 6–8) than for vehicle (lane 5). Somewhat analogous to the changes in retinal rod cell proteins, the levels of cone cell opsin and cone cell arrestin (mCAR) decreased in extracts from rats treated with 24 h of intense light when compared with 4 h light-treated rats (lanes 5–8) or those unexposed to light (lanes 1–4). In the 4-h light-treated rats, zinc and/or rosemary treatment led to a small increase in the presence of cone opsin, with no apparent increase in mCAR levels.

### Rosemary oil diluted in ROPUFA

**Figure 5:**
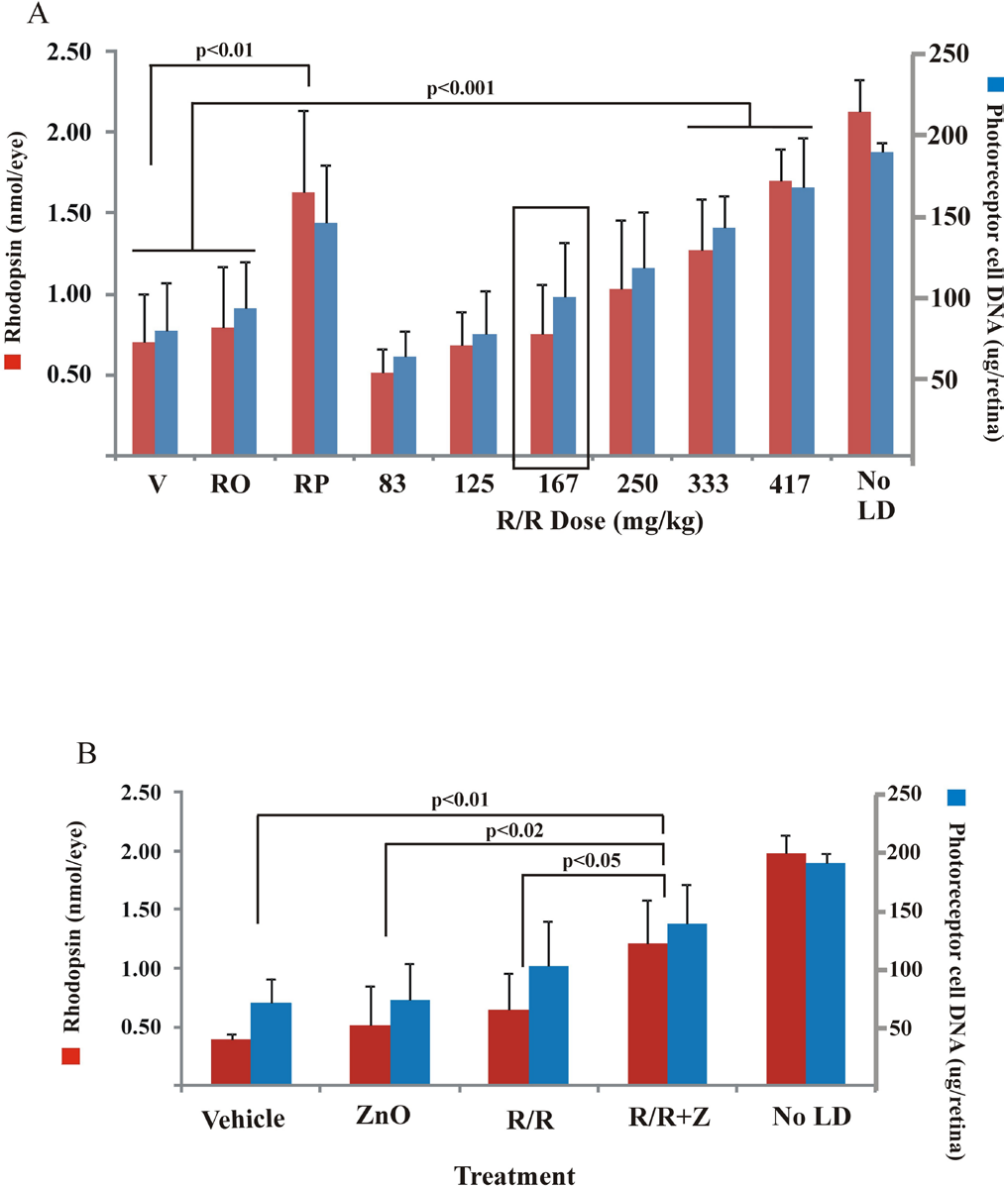
Visual cell survival in light-exposed rats given rosemary oil extract. **A**: Dark-reared rats were treated with different volumes of rosemary oil extract dissolved in a commercial mixture of polyunsaturated fatty acids (ROPUFA) and then exposed to 4 h of moderate intensity green light. Rhodopsin and photoreceptor cell DNA were measured 2 weeks later. A nearly linear increase in visual cell survival occurred with increasing doses of rosemary in ROPUFA (R/R). For both the 333 and 417 mg/kg doses, rhodopsin and DNA levels were significantly higher than for the Tween-80 vehicle (V) or ROPUFA (RO) alone. These values were nearly the same as for a detergent extract of rosemary powder (RP) given at 34 mg/kg bodyweight. No LD represents data from rats unexposed to intense light; n=4-8 rats per dose, ±standard deviation. **B**: Rats were treated with rosemary oil plus zinc oxide and then exposed to intense white light. Rosemary oil (167 mg/kg) alone (R/R), zinc oxide (0.65 mg/kg, ZnO) alone, or rosemary oil plus zinc oxide (R/R+Z) was given to rats 1 h before intense light exposure. Rhodopsin and DNA were measured as described in the Materials and methods section. At the doses used neither R/R nor ZnO was effective when compared to vehicle treatment. Visual cell survival for R/R+Z-treated rats, however, was significantly higher than for vehicle, ZnO, or R/R alone. Values for rhodopsin and DNA in vehicle-treated rats exposed to light are the average for 1% Tween-80/10% ethanol, ROPUFA, and acidified water vehicles. For these experiments there were n=4-5 rats per dose and the data is presented ±standard deviation.

Rosemary powder is essentially 100% rosemary prepared by extraction, blending, and drying such that the antioxidant carnosic acid is present in excess of the other actives (Appendix 1). Rosemary oil extract contains those same actives but is often diluted with triglycerides or phospholipids to preserve antioxidant efficacy. Here we used rosemary oil extract diluted with ROPUFA, a mixture of long-chain polyunsaturated fatty acids, primarily ethyl esters of eicosapentaenoic (EPA) acid and DHA. Figure 5A shows that the rosemary–ROPUFA mixture (R/R) was effective in promoting photoreceptor cell survival following intense light treatment. For rats given increasing doses of R/R, we found a linear increase in the levels of rhodopsin and retinal DNA. For the two highest doses, rhodopsin and DNA levels were significantly higher than for the 1% Tween-80/10% ethanol vehicle or for vehicle containing only ROPUFA (p<0.001). Comparisons of retinal DNA in light-exposed rats revealed that a tenfold higher dose of rosemary oil extract (333 mg/kg) was approximately as effective as a 34-mg/kg dose of rosemary powder. Analysis of the carnosic acid contents in R/R and rosemary powder at those doses indicated that the oil extract contained about three times more carnosic acid than the powder (Appendix 1). Previously we found that a rosemary oil extract solubilized with lecithin was equally effective when given to rats at a dose of 327 mg/kg [16].

Using a single dose of R/R with approximately 50% protective activity (Figure 5A, boxed), we tested it in combination with zinc oxide in light-exposed rats. As shown in Figure 5B, neither 167 mg/kg of the R/R extract nor 0.65 mg/kg of aqueous zinc oxide was very effective. The average levels of rhodopsin and DNA were only slightly higher than for vehicle. However, the combination of rosemary plus zinc (R/R+Z) was much more effective than either component alone. Rhodopsin and DNA levels were 60%–70% of the levels in unexposed control rats and significantly higher than in light-exposed animals given vehicle (p<0.01), those given zinc alone (p<0.02), or those given only R/R (p<0.05).

### White light exposure

**Figure 6:**
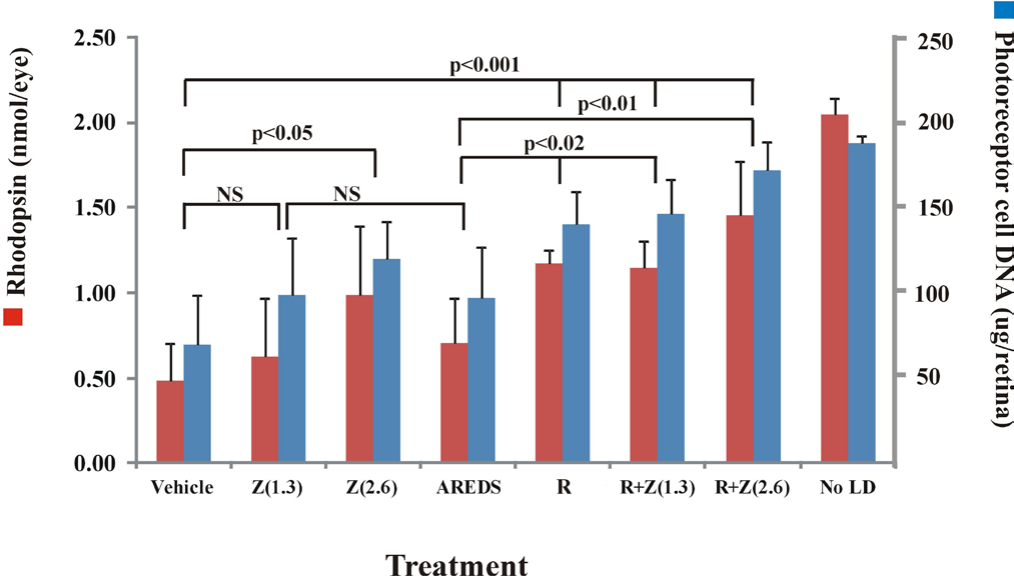
Protection from retinal light damage by rosemary with or without zinc oxide versus treatment with the antioxidant/zinc combination recommended by the Age Related Eye Disease Study group (AREDS1). Cyclic light-reared rats were treated with zinc oxide (Z; 1.3 or 2.6 mg/kg), rosemary powder extract (R; 34 mg/kg), or both (R+Z) and then exposed to full-spectrum intense white light for 6 h. Rhodopsin and retinal DNA were measured 2 weeks later to assess visual cell survival. Rosemary treatment and rosemary plus zinc were significantly more effective than treatment with vehicle (p<0.001). Other rats were treated 1 h before light exposure with an AREDS antioxidant–mineral mix given at approximately the daily recommended dose for zinc (1.3 mg/kg). AREDS treatment was ineffective when compared with either light-exposed vehicle-treated rats or rosemary and rosemary plus zinc treatment (p<0.02 or p<0.01, respectively). Values shown are the averages for n=5–12 rats ±standard deviation. NS is equal to no significant difference.

Moderate intensity green light effectively bleaches rhodopsin and initiates retinal light damage [31], but higher intensity, full-spectrum, white light more effectively mimics normal environmental lighting. Accordingly, we determined the effectiveness of zinc and rosemary treatment in cyclic light-reared rats, which are more resistant to light damage than dark-reared animals (20), following exposure to 9,000 lux white light for 6 h. As shown in Figure 6, increasing the concentration of zinc oxide from 1.3 to 2.6 mg/kg resulted in a significant increase in the levels of rhodopsin and retinal DNA (p<0.05 versus vehicle). Rosemary alone was more effective than zinc oxide; rhodopsin and DNA levels were 60%–70% of the values found in animals unexposed to intense light. These values were also significantly higher than for vehicle-treated rats exposed to light (p<0.001). Combined treatment with rosemary plus zinc was even more effective. For rosemary plus 2.6 mg/kg zinc oxide, rhodopsin and retinal DNA levels were 70% and 90%, respectively, of those in unexposed control rat retinas. Visual cell survival after combined treatment, at either the high or low dose of zinc oxide, was also significantly higher than for rats treated with vehicle and intense light (p<0.001).

The efficacy of an AREDS1 antioxidant mineral mixture [12] was compared to that for a rosemary powder extract (34 mg/kg) with or without a comparable dose of zinc oxide. Figure 6 shows that this AREDS antioxidant preparation was ineffective in preventing light-induced retinal damage. Although average rhodopsin and DNA levels were higher than for vehicle-treated rats, the differences were not significant. However, rosemary alone and rosemary combined with 1.3 mg/kg of zinc oxide effectively decreased the extent of retinal light damage. The levels of rhodopsin and photoreceptor cell DNA were significantly higher than for treatment with the AREDS vitamin/mineral mix (p<0.02). When the same dose of rosemary was combined with a higher level of zinc oxide (2.6 mg/kg), retention of rhodopsin and retinal DNA was also significantly higher (p<0.01) than for AREDS treatment.

### Retinal protein electrophoresis

**Figure 7:**
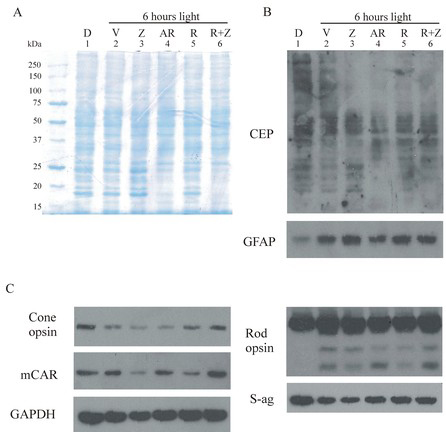
Western analysis of retinal proteins following intense white light treatment. Coomassie staining of retinal proteins, with 20 micro grams protein per lane (**A**), western analysis of oxidative protein markers (**B**), and the retention of visual cell opsins and arrestins (**C**) in retinas from rats treated with six hours of intense white light. Light exposed rats, n=4 per treatment, were given zinc oxide (Z, 1.3 mg/kg), a detergent extract of the Age Related Eye Disease study group (AREDS) vitamin-mineral mix (AR) containing 1.44 mg/kg zinc oxide, rosemary (R, 34 mg/kg), or rosemary plus zinc oxide (R+Z) and compared with vehicle treatment (V) or no light exposure (D). **A**: Protein staining was relatively uniform, but increased levels of low molecular weight proteins were seen in lanes 1, 2, 3, and 5. **B**: Antioxidant treatment (lanes 3–6) reduced the level of carboxyethylpyrrole (CEP) staining for high molecular weight proteins, while AR and R+Z treatment (lanes 4, 6) also resulted in reduced staining for low molecular weight proteins. Intense light exposure led to an increase in the level of the Muller cell oxidative stress marker glial fibrillary acidic protein (GFAP) in all samples (lanes 2–6). **C**: R+Z treatment was more effective than AR in preserving cone cell opsin and cone arrestin (mCAR) immunoreactivity. Modest degradation of rod cell opsin and S-antigen, rod arrestin (S-ag) occurred in all light-exposed rat retinal samples. GAPDH staining, used as an indicator of protein loading, was slightly reduced in lane 3.

Retinal proteins from rats treated with vehicle, zinc oxide (1.3 mg/kg), AREDS vitamin/mineral mix at a nearly comparable zinc level, rosemary (34 mg/kg), or rosemary plus zinc (R+Z), followed by exposure to intense white light were compared with those from rats unexposed to light (Figure 7). Coomassie staining showed a relatively uniform distribution of retinal proteins among all treatment groups (Figure 7A). However, light-exposed AREDS and R+Z-treated rats (lanes 4, 6) had reduced levels of low molecular weight proteins (<25 kDa) compared to vehicle, zinc, or rosemary alone (lanes 2, 3, 5).

Western analysis of the oxidation marker CEP (Figure 7B) showed a range of CEP-adducted proteins of various molecular weights for rats unexposed to light (lane 1) and for those given vehicle and treated with intense white light (lane 2). Zinc oxide treatment (lane 3) resulted in increased CEP reactivity for proteins <25 kDa, which correlated with the protein staining pattern in Figure 7A. However, all antioxidant treatments (lanes 3–6) led to reduced CEP immunostaining for proteins >75 kDa. AREDS and treatment with R+Z (lanes 4, 6) also resulted in reduced levels of staining for proteins <25 kDa. The Muller cell stress protein marker GFAP increased markedly in all light-treated rat retinas, with slightly less staining for AREDS and R+Z.

Cone cell opsin immunoreactivity was dramatically reduced by intense light exposure of rats treated with vehicle (lane 2), those given zinc oxide (lane 3), or those treated with AREDS (lane 4; Figure 7C). Rosemary treatment and R+Z (lanes 5, 6) resulted in higher levels of cone opsin, with staining for R+Z treatment appreciably greater than for AREDS. R+Z treatment also resulted in more intense mCAR reactivity than seen for AREDS or for the other antioxidants. Staining for the monomer of rod cell opsin was intense in all samples and did not appear to be appreciably affected by intense light. However, as shown by two lower molecular weight opsin fragments, degradation was clearly occurring 48 h after exposure. The pattern of immunostaining for rod cell S-ag did not exhibit low molecular weight degradation products, but intense light did result in a slight reduction in S-ag levels for all treatment groups.

## Discussion

This study shows that zinc oxide and extracts of the herb rosemary both prevent the loss of retinal photoreceptor cells arising from intense light-induced oxidative damage and that protection was greater with their combination than for either treatment alone. Visual cell survival was enhanced by zinc oxide combined with either a detergent extract of rosemary powder (Figure 1) or rosemary oil diluted with ROPUFA (Figure 5). The protective effect was seen for dark-reared rats subsequently exposed to moderate intensity green light and for cyclic light-reared animals treated with higher intensity, full-spectrum, white light (Figure 6). Enhanced photoreceptor cell survival from zinc oxide plus rosemary was confirmed by improved retinal morphology (Figure 2) and by reduced DNA fragmentation (Figure 3) compared with vehicle treatment. Using western analysis we also found increased levels of visual cell transduction proteins and reduced levels of oxidative protein markers (Figure 4 and Figure 7), further supporting the enhancement of photoreceptor cell survival, at least in part, by an antioxidant effect. Finally, the retention of cone cell opsin and mCAR was improved by the combined zinc and rosemary treatment. This indicates that cone photoreceptors are also affected by acute light-induced oxidative stress and confirms earlier work showing that oxidative stress can impact cone survival in late-stage retinal disease [34].

Traditional antioxidants in combination with zinc are present in the AREDS vitamin/mineral mix, and an antioxidant combination has recently been shown to prevent light-induced RPE cell death [35]. In this study we used a widely available commercial AREDS preparation [12], which contains identical amounts of vitamins and minerals to those tested in the clinical trial, and on a mg/kg basis approximates the mean levels for men and women, see Appendix 1. However, in our study the AREDS antioxidant–zinc combination proved to be ineffective, while the rosemary–zinc combination effectively reduced oxidative stress (Figure 4 and Figure 7B) and prevented retinal light damage (Figure 1, Figure 5 and Figure 6). This suggests that the protective efficacy for the phenolic antioxidants in rosemary [17-20] is greater than for the more traditional antioxidants present in a typical AREDS1 formulation and that augmentation of AREDS with rosemary may have a further positive impact on visual cell survival during retinal degeneration.

Oxidative stress has been implicated in the mechanism of light-induced retinal degeneration [15,36] and as part of the etiology of AMD [4,8,12,14]. Drusen isolated from AMD eyes contains higher levels of oxidized lipid–protein adducts than found in eyes from unaffected age-matched individuals [10]. Among these, CEP is specifically formed from oxidation of DHA and is known to form lipid–protein adducts in vivo [10,22]. In this study we found decreases in retinal protein–CEP reactivity following antioxidant treatment of rats exposed to intense white light (Figure 7). This indicates that antioxidants can impact the formation of at least some CEP adducts under oxidative stress conditions. Intense white light also resulted in losses of cone opsin and mCAR, but AREDS treatment was less effective than rosemary with zinc in preventing their loss, suggesting that the combination of rosemary plus zinc was more efficacious. Previously, Hollyfield et al. [26] showed that treatment of mice with CEP adducted to mouse serum albumin induced inflammation and caused modifications to Bruch’s membrane that resemble those seen in AMD. DHA and its 20:5 n-3 precursor EPA are present in relatively high amounts in fish oil and may decrease both the incidence of AMD [37] and the presence of advanced disease [38]. In our acute light-damaged animal model, ROPUFA alone, which contains 20% DHA and 38% EPA, was ineffective (Figure 5). It remains to be determined if long-term dietary administration of ROPUFA would increase its protective efficacy in this animal model.

In experimental animals, light-induced retinal degeneration has been found to activate the immune complement system and to increase deposition of these proteins at the sites of retinal damage [28,29]. CFH is a major complement protein, now known to bind oxidized proteins in serum or on cell surfaces and to protect against continuation of an oxidative cascade and tissue damage [14]. At micromolar levels, zinc ion potentiates CHF binding to factor C3b [13], thereby protecting from a complement-induced progression of oxidative damage. The use of more effective antioxidants, therefore, should allow lower concentrations of zinc to be used clinically, enhancing both its beneficial effect on CFH and reducing its potential toxicity [39,40]. In this regard it is noteworthy that a lower dose of zinc oxide is currently being tested in one arm of the AREDS2 clinical trial [41].

Rosemary contains a complex mixture of phenolic and di- and tri-terpenoid water-insoluble antioxidants, with relatively large amounts of carnosic acid, carnosol, and ursolic acid as well as several other compounds in lower quantities [17-20]. The levels of antioxidants, their stability, and their efficacy all depend on the source of rosemary, their solubility, the suspension media, and environmental factors, such as light and temperature. For example, n-3 fatty acids, such as found in ROPUFA, can stabilize carnosic acid and prolong its half-life considerably [20]. Recently, carnosic acid has been shown to induce phase II antioxidant enzymes in vitro and to prevent oxidative stress in RPE and W661 cells [42]. Chronic administration of carnosic acid (25 mg/kg/day) also appears to prevent retinal light damage in rats [42]. In our experiments a single dose of rosemary extract given 1 h before intense light effectively reduced visual cell damage and loss, especially when combined with zinc oxide. While rosemary oil extract in ROPUFA was equally effective, it also contains about threefold more carnosic acid and twofold more ursolic acid than our rosemary powder extracts (Appendix 1). It remains to be determined which component(s) of rosemary might induce antioxidant enzymes and influence transcription in vivo or if they act by direct interaction with reactive oxygen species or both to decrease oxidative damage and improve cell viability.

Animal models cannot possibly capture all aspects of human ocular disease, and a note of caution regarding interpretation of these experiments seems appropriate. Extrapolation of results from rodent retinas to those of primates is limited by major anatomic differences in rod and cone cell distributions [25] and by circadian differences between species [21]. Despite these differences, end-stage retinal remodeling following extensive visual cell loss, in either advanced atrophic AMD or in the light-damaged rat retina, is remarkably similar [30]. Acute experimental light-induced retinal degeneration has long served as a preclinical model for oxidative stress [15,36], and our results suggest that it can also serve as a relatively rapid and inexpensive model for comparing drug treatments or antioxidant efficacy. In this acute light-damaged rat model, AREDS was significantly less effective than a rosemary extract plus zinc in reducing rod photoreceptor cell loss and in preserving cone cell opsin and arrestin. It is tempting to speculate about the implications of this improvement in antioxidant efficacy from these results. However, AREDS is normally recommended for long-term treatment, and as such has been shown to decrease the rate of disease progression in approximately 25% of patients with advanced AMD [12]. Similarly, diabetic rats treated chronically with an AREDS-based antioxidant diet were shown to exhibit a reduction in the progression of retinal pathology [23,24]. We are currently testing chronic AREDS and zinc oxide administration with and without rosemary extract in our rat model of light-induced retinal degeneration to determine if the model can effectively mimic outcomes expected from earlier and current AREDS clinical trials.

## Appendix 1. Prevention of retinal light damage by zinc oxide combined with rosemary extract Analysis of Actives.

To access the data, click or select the words “Appendix 1.”
